# Alginate–Halloysite Nanocomposite Aerogel: Preparation, Structure, and Oil/Water Separation Applications

**DOI:** 10.3390/biom10121632

**Published:** 2020-12-03

**Authors:** Sneha Bhagyaraj, Igor Krupa

**Affiliations:** Center for Advanced Materials, Qatar University, P.O. Box 2713, Doha 2713, Qatar

**Keywords:** alginate aerogel, halloysite, nanocomposite, oil/water separation

## Abstract

Environmental remediation using green approaches for addressing various pollution-related issues, especially water pollution, is in high demand. Here, we designed an environmentally friendly, low-cost, and stable sodium alginate–halloysite clay composite aerogel (SAHA) for oil/water separation via a two-step synthesis procedure, including ionic crosslinking and freeze-drying. The as-prepared SAHA aerogels were characterized in detail by scanning electron microscopy (SEM), atomic force microscopy (AFM), X-ray diffraction (XRD), and Fourier transformation infrared (FT-IR) spectroscopy. Characterization of the SAHA aerogels revealed a three-dimensional porous microstructure with uniformly dispersed halloysite nanotubes (HA) within the alginate matrix. The elemental composition of the hydrogels investigated using energy dispersive X-ray spectrometry (EDX) revealed the presence of minerals, such as magnesium, sodium, aluminum, and silicon in the SAHA aerogels. The presence of a hydrophilic alginate matrix combined with these unique morphological characteristics resulted in SAHA aerogels with underwater oleophobicity and excellent oil/water separation efficiency (up to 99.7%). The ease of fabrication, excellent oil/water separation, and multiple performances make the SAHA aerogel an interesting candidate for practical applications in water recycling.

## 1. Introduction

Aquatic pollution caused by frequent oil spills or contaminants from industries, such as petrochemical, food, textile, and steel industries, has severely affected marine ecology as well as human beings. Developing effective environmentally friendly approaches to tackle oil spill pollution problems has attracted great interest among the scientific community. Numerous methods, such as filtration, absorbents, skimmers, biodegradation, and in situ combustion, have been successfully used to remove oil spills [1,2,3,4,5]. One of the most effective techniques for the separation of oil/water mixtures is the use of filter materials with specific wettability to separate oil directly from oil/water mixtures without any other external forces [6]. Polymer composite membranes, particularly electrospun nanofiber membranes have been often explored for an effective separation of oil/water mixtures [7,8,9]. Three-dimensional, highly porous structures, such as aerogel foams with extremely low density, are widely used as effective absorbents for oil/water mixtures [10]. To make these foams superhydrophobic for oil absorption, various treatments, such as silane coupling or sulfonation, are carried out, which further increases the cost of production [11]. The superhydrophobic property of the foams drives the oil absorption capacity, which further results in blocking of the pores, thereby decreasing the efficiency of absorption. Aerogel foams can also be used as filters for gravity-driven oil/water separation with high flux [12].

Alginate, derived from brown sea algae, is a renewable, low-cost, environmentally friendly, linear polysaccharide. Being an abundant biopolymer and less toxic, it has been extensively studied for various applications, especially in biomedical research, drug delivery, pervaporation, and membrane filtration [13,14,15]. The swelling and degradation behavior of alginate in water limits the application of alginates in various industrial applications. To minimize swelling and improve the mechanical properties of aerogels, various techniques, such as crosslinking, adding fillers, and functionalization, are used [16,17]. Recent studies have proven that alginate possesses excellent salt-tolerant and underwater superoleophobic properties and therefore shows great potential to achieve oil/water separation in marine environments [18]. However, the results from some studies revealed that the performance of alginate–based aerogels decreases gradually with the number of reuses; therefore, alginate–based aerogels require extensive attention, and further research is necessary to improve the separation efficiency and service life [19].

Halloysite clay is a volcanic-derived mineral consisting of layered aluminosilicate with a high surface area and aspect ratio [20]. It is a product of basalt weathering with the formula Al_2_Si_2_O_5_(OH)_4_. When dry, the mineral is very brittle, and under very low loads, it easily disintegrates into irregular crumbs. Similar to other kaolinite minerals, it adsorbs water, but unlike montmorillonite, this water adsorption does not lead to an increase in volume (swelling effect). After drying at temperatures above 60 °C, halloysite loses water, and its structure becomes similar to kaolinite with a heterogeneous disordered structure. Nanocomposites incorporating clay minerals have become increasingly promising because of the reinforcement effect of the nanocomposite due to the presence of the intercalated structure of clay [21]. Halloysite clay has been studied as a filler for designing of composites for various applications such are cosmetics, electronics, catalysis, etc., due to its abundance, biocompatibility, and low toxicity [22,23,24]. Recently, Zhao et al. prepared chitin/ halloysite nanotube (HNT) composite sponges modified by bromohexadecane in order to enhance their hydrophobicity for oil–water separation with a separation efficiency as high as 98.7% [25]. In another study, Song et al. prepared a modified cotton fabric with strong water-resistance and high oil/organics affinity using octadecyl-trimethoxysilane-modified HNTs [26].

Alginate–halloysite composite structures have shown promising characteristics, which are useful in various fields including drug delivery, catalysis, and as an effective absorbent of various dyes [27,28]. Liu et al. reported the preparation and in vitro evaluation of a series of alginate/halloysite nanotube (HNTs) composite scaffolds with improved mechanical properties and cell attachment ability [29]. Huang et al. studied the effect of HNTs on the physical properties of alginate aerogels with improved dynamic storage modulus [30]. Recently, sodium alginate–halloysite nanotube gel beads were reported as potential candidates for delivering anticancer compounds [31]. To the best of our knowledge, there is no report regarding the use of alginate–halloysite composite aerogels for oil/water separation applications. In this report, we synthesized a sodium alginate–halloysite clay nanocomposite aerogel by using a two-step ionic crosslinking and freeze-drying technique. The structural and chemical characteristics are studied in detail. Furthermore, the effect of halloysite clay nanotubes on the oil/water separation efficiency of the aerogel is also evaluated.

## 2. Materials and Methods

### 2.1. Materials

Sodium alginate (SA) (alginic acid sodium salt from brown algae), pure anhydrous calcium chloride (CaCl_2_), and halloysite nanoclay (kaolin clay) were acquired from Sigma-Aldrich, China. The reagents were used as received without any purification. The oils included corn oil (0.917 g/cm^3^) (Cornlite, Nashik, India), hexane (0.659 g/cm^3^) (Sigma-Aldrich, Shanghai, China), and pump oil (0.880 g/cm^3^) (Mobil, Surrey, UK).

### 2.2. Preparation of Sodium Alginate Aerogel (SA)

Sodium alginate stock solutions were prepared by dissolving an appropriate amount of sodium alginate powder in demineralized Milli-Q water, followed by homogeneous mixing by mechanical agitation using a homogeneous mixer at room temperature for 4 h. To prepare 2 wt. % sodium alginate solutions, 2 g sodium alginate powder were added in 100 mL water followed by described procedure to disperse it into a solution. The as-prepared homogeneous solution of sodium alginate was poured into a round petri dish (1 cm thickness and 8.5 cm diameter) followed by 30 min drying in a vacuum oven to degas the solution. Then, the casted solution was maintained at −80 °C overnight followed by freeze-drying at –54 °C for 48 h. The as-prepared sodium alginate aerogel was immersed in 3 wt. % CaCl_2_ solution for 24 h to allow for the crosslinking process to occur. After ionic crosslinking, the aerogel was washed thoroughly to remove excess chloride ions and then freeze-dried again to obtain the final sodium alginate aerogel.

### 2.3. Preparation of Sodium Alginate–Halloysite Nanocomposite Aerogel (SAHA)

In a typical synthesis, 0.1 g halloysite clay (HA) was dispersed in 100 mL water using mechanical stirring followed by ultrasonic mixing to obtain a uniform solution. To the as-obtained homogeneous solution, 2 g SA was added followed by vigorous stirring to obtain a uniform SA-HA hybrid sol. The as-prepared sol was cast into a petri dish (1 cm thickness and 8.5 cm diameter) and kept overnight at −80 °C. Freeze-drying was carried out for 48 h to obtain an uncrosslinked SA-HA aerogel. The aerogel was then immersed in 3 wt. % CaCl_2_ solution overnight to initiate the crosslinking process, followed by freeze-drying of the crosslinked SA-HA composite aerogel to obtain the final product, a sodium alginate–halloysite clay composite aerogel (SAHA).

### 2.4. Characterization

Morphological characteristics were evaluated using field emission scanning electron microscopy (FE-SEM, Hitachi SU8000) with an accelerating voltage of 5 kV after sputter-coating with gold under vacuum. Atomic force microscopy (AFM) (Asylum Research UK-MFP3D, scanning mode and Nano Indentation) were used to quantify the surface roughness. Transmission electron microscopy (TEM) was performed using a JEOL JEM-3010 electron microscope (Japan) operating at 200 kV. X-ray diffraction analysis was performed to analyze the crystalline structures using a diffractometer (PANalytical model X’PERT-PRO, Malvern, UK) with K_α_ radiation of 1.5418 Å and a scan rate of 10°/min. To analyze the chemical structure of the aerogel, FT-IR was performed using a PerkinElmer Spectrum 400 spectrophotometer (Waltham, MA, USA) in the range 400–4000 cm^−1^ with a resolution of 2 cm^−1^. The specific surface area and pore size of the alginate aerogels were determined by Brunauer- Emmett-Teller (BET) analysis using an ASAP-2020 Micrometrics surface area and porosity analyzer, USA. The water and oil contact angles were measured using a drop shape analysis system (SCA-20U, Data physics instruments, Filderstadt, Germany) by dispensing 2 μL of water or oil droplets onto the aerogel surfaces. Five different locations on the surfaces were examined to obtain an average value. Thermal studies of the samples were performed by thermogravimetric analysis (TGA 4000, Perkin Elmer, Greenville, SC, USA) in the temperature range from 30 to 800 °C at a heating rate of 10 °C/min under a nitrogen atmosphere with approximately 10–15 mg of sample for each experiment.

#### 2.4.1. Porosity Measurement

The average porosity of the aerogel was determined by a fluid replacement method [32]. The average porosity (P) was calculated using Equation (1).

P = (V_p_/V_s_) × 100 (1)

where V_p_ is the pore volume measured using the ethanol displacement method, and vs. is the geometrical volume of the sponge calculated using diameter and height. The pore volume V_p_ was calculated using the equation

V_p_ = (W_e_ − W_o_)/ρ_e_V_s_ (2)

The aerogel with an initial weight of W_o_ (initial sponge) was immersed in absolute ethanol at room temperature and then placed in a desiccator under reduced pressure for 5 min to remove air bubbles. After gently removing surface ethanol with filter paper, the samples were weighed immediately as W_e_; ρ_e_ is the density of ethanol (0.789 g/cm^3^). Average values were obtained from five replicates for each sample. Ethanol was chosen as the displacement liquid, because it penetrates easily into the pores without inducing any shrinkage or swelling.

#### 2.4.2. Oil–Water Separation Test

The oil/water separation experiment was carried out using a homebuilt setup. Before the oil/water separation experiment, the SA and SAHA aerogels (5 × 2.5 × 0.25 cm size) were saturated with water and then placed in the center of a glass funnel. The funnel was then placed on top of a graduated cylinder to measure the filtrate directly throughout the experiment, which was driven by gravity. A 40 mL oil–water mixture (1:1 by volume) was used in this study. The mixture was poured into the funnel containing the aerogel, and the system was kept still for 2 min, followed by measuring the volume of water collected to evaluate the oil/water separation efficiency of the aerogel. Three types of oils and organic solvents, including corn oil, hexane, and vacuum oil, were used in the experiment.

The separation efficiency ŋ was calculated by Equation (3):

ŋ (%) = (V_c_/V_0_) × 100% (3)

where V_0_ and V_c_ represent the initial and collected water volumes, respectively.

Oil intrusion pressure measurement. The oil intrusion pressure measurement was also conducted with the homebuilt setup. The aerogels (5 × 2.5 × 0.25 cm size) were saturated with water and placed inside one end of a long glass tube. Then, the glass tube was mounted onto another glass cylinder, so that the aerogel sample separated the two glass tubes. In the test, pure corn oil was poured slowly into the upper glass tube. As the volume of the oil increased, the oil pressure on the aerogel surface also increased, and finally, when the pressure exceeded the critical value, oil droplets began to penetrate the aerogel. The maximum height of the oil was denoted as h_max_. The oil intrusion pressure (P) was calculated using Equation (4):

P = ρ × g × h_max_ (4)

where ρ represents the density of corn oil (~0.93 g/cm^3^), g is the acceleration of gravity, and h_max_ is the maximum height of the oil column that the aerogel can support.

## 3. Results and Discussion

**Figure 1:**
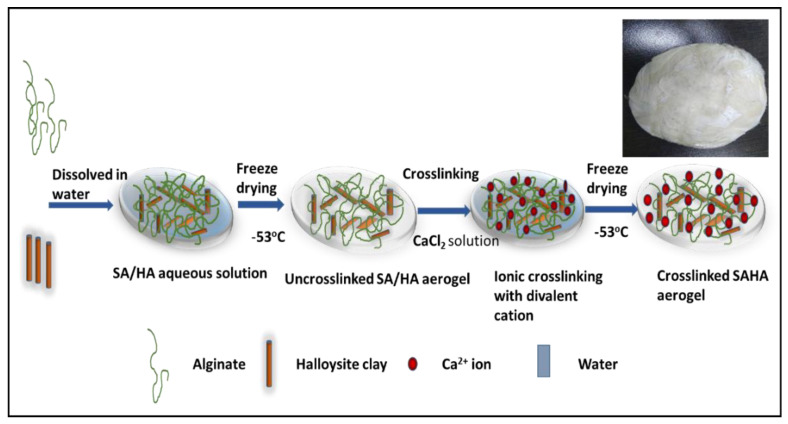
Schematic representation of the preparation of the sodium alginate–halloysite composite aerogel (inset: digital image of the as-prepared sodium alginate–halloysite (SAHA) aerogel).

Studies have revealed that directly introducing divalent ions into alginate solution can result in the formation of anisotropic gel particles instead of homogenous gel because of the rapid crosslinking reaction between the carboxyl group of alginate and the divalent ions [33]. Hence, in this study, a two-step freeze-drying process was employed for the preparation of SA and SAHA aerogels. Initially, SA alone was freeze-dried, followed by crosslinking of the alginate using CaCl_2_ solution. Then, the crosslinked calcium alginate membrane was freeze-dried to form the corresponding aerogel. The same steps were followed for the preparation of the SAHA aerogel except for the addition of halloysite nanoclay tubes to the alginate solution. A schematic representation for the preparation of the SAHA aerogel and a digital image of the product are shown in Figure 1.

### 3.1. Determination of Morphological Properties

#### 3.1.1. X-ray Diffraction Study

**Figure 2:**
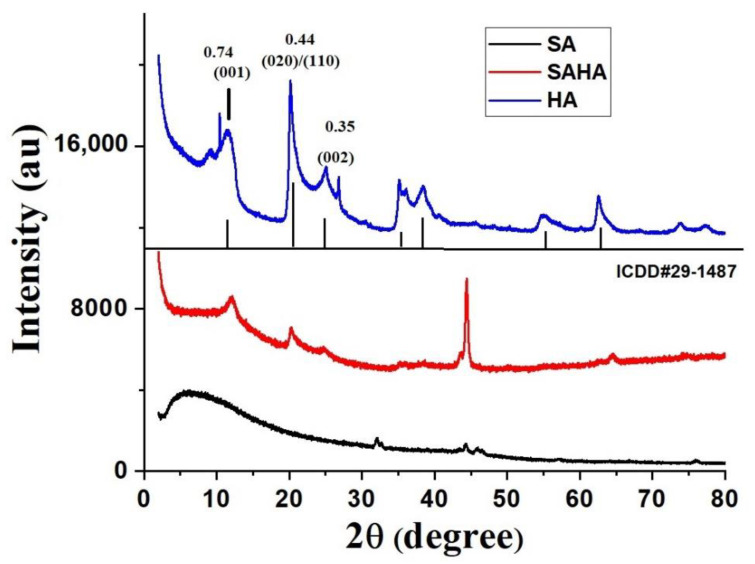
XRD patterns of the as-prepared alginate–halloysite composite aerogels.

Figure 2 shows the X-ray diffraction pattern of the as-synthesized SA and SAHA aerogels and HA. Alginate, a semicrystalline polymer, shows no significant peaks in the pattern. In the pattern of halloysite clay, significant peaks can be observed, which represent the minerals in the clay sample. The sharp peaks at 24.32 and 25.62° are attributed to the 100 reflections of the quartz impurity (ICDD file no. 33–1161) and the 101 reflection of cristobalite (ICDD file no. 39–1425), respectively. The reflections at 12.0° (001) correspond to a basal spacing of 0.74 nm, while that of 20.4° (020)/(110) corresponds to 0.44, respectively [34]. The XRD spectrum is fully consistent with halloysite of basal spacing 7.3 A° (ICDD file no 29–1487) [35]. The absence of a peak at 8.8° (2θ) represents the basal spacing of hydrated halloysite (10 A°) [36]. Compared to the SA aerogel, the SAHA aerogel showed multiple diffraction peaks attributed to kaolin clay. Meanwhile, in the case of SAHA aerogel, a peak at 44° can be seen, which may be due to some defect associated with the carbon entity present [37].

#### 3.1.2. Transmission Electron Microscopy (TEM) Analysis

**Figure 3:**
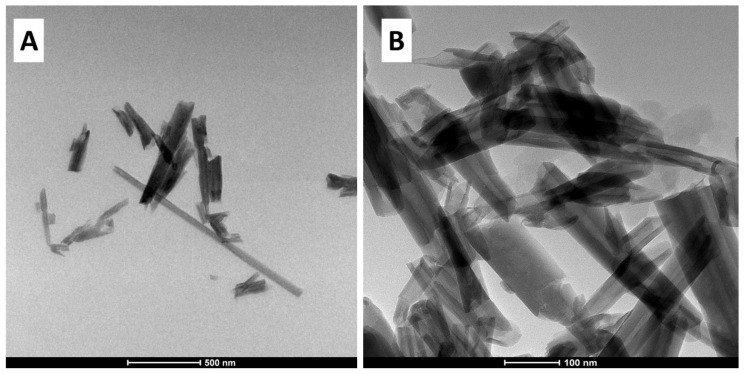
TEM images of (**A**,**B**) halloysite clay (HA) at different magnifications.

The morphology of the halloysite nanoclay tubes was analyzed using TEM, as shown in Figure 3A,B. The image revealed that the HA are tubular, cylindrical shaped and possess an open-ended lumen. The average outer diameter of nanotubes found to be 40−80 nm and an inner lumen diameter 10−20 nm, together with a wall thickness of around 25 nm.

#### 3.1.3. Scanning Electron Microscopy (SEM)

**Figure 4:**
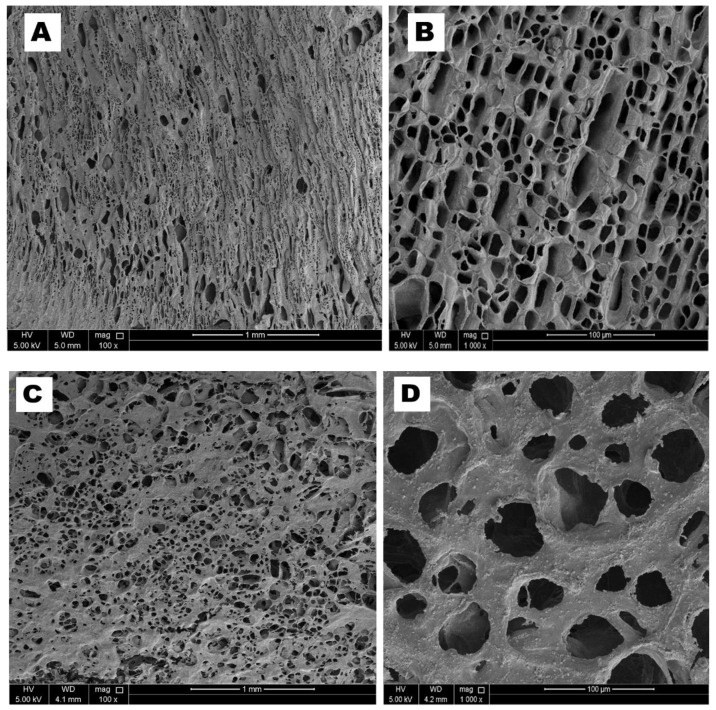
SEM images of the as-prepared (**A**,**B**) 2% sodium alginate aerogel (**C**,**D**) SAHA aerogel with different magnifications.

A morphological examination of the as-prepared aerogels was performed by FE-SEM analysis, and the images with different magnifications are shown in Figure 4. The images reveal a well-connected 3D porous structure for all aerogels. FESEM images for the 2 wt. % sodium alginate aerogel are shown in Figure 4A,B. The SA aerogel showed honeycomb-like porous morphological characteristics with randomly distributed pores with pore sizes in the range of 40–150 μm. Previous studies have already recommended that, when using aerogels for oil/water separation applications, the pore size should be below 200 μm [38]. Hence, 2 wt. % SA solution was used in this study for preparing the nanocomposite aerogels. Figure 4C,D show the images for the as-prepared SAHA composite aerogel at different magnifications. A pore size analysis of the aerogel using image analysis software (ImageJ) revealed randomly distributed pores in the range of 50–180 μm. From the high-resolution SEM image (Figure 4D), the pores in the aerogels appear to be irregular with a rough surface. The increase in pore size of the SAHA aerogel can be attributed to the formation of an internal network structure via hydrogen bonding between the halloysite clay and the sodium alginate matrix [39]. This unique pore feature due to the highly distributed network structure formed inside the aerogel allows for a gravity-driven oil/water separation process with high flux. The surface roughness of this water-soluble polymer plays a significant role in obtaining underwater superoleophobic properties of the aerogels. The percentage porosity calculated using the displacement method for the sodium alginate aerogel was 86.4 ± 0.7, whereas that for the SAHA aerogel was 77.9 ± 0.5. The percentage porosity of the composite aerogel decreases compared to the neat polymer aerogel, since the solid content in the composite aerogel precursor is more.

#### 3.1.4. Atomic Force Microscopy Analysis (AFM)

**Figure 5:**
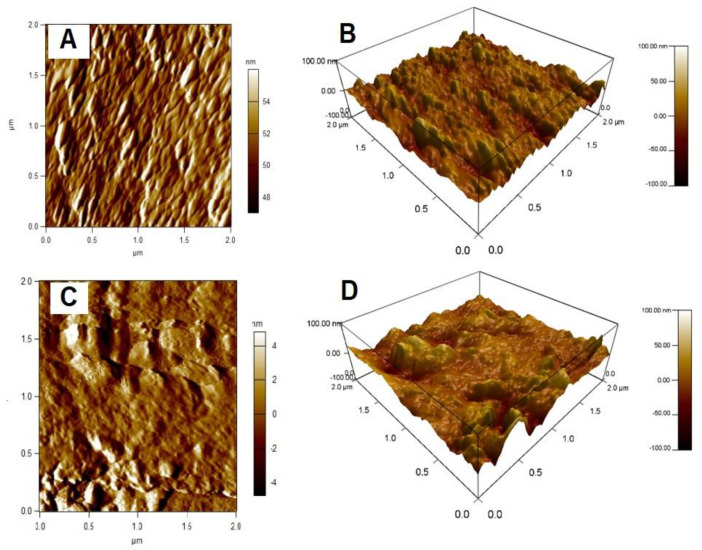
Atomic force microscopy (AFM) images (**A**,**C**) amplitude retrace mode and (**B**,**D**) 3D images of SA and SAHA aerogels, respectively.

The AFM images of the sodium alginate aerogel (SA) and the composite sodium alginate–halloysite clay nanotube aerogels (SAHA) are shown in Figure 5. SA aerogel shows a relatively smooth surface (Figure 5A) when compared to that of SAHA aerogel (Figure 5C) in their corresponding amplitude trace scan mode images. From the 3D images, it is very clear that the SAHA aerogel (Figure 5D) is having more uneven surfaces with major crest and trough features indicating that the surface roughness is increased compared to that of SA (Figure 5B).

#### 3.1.5. BET Surface Analysis

As shown in Table 1, which represents the values obtained from the Brunauer- Emmett-Teller (BET) surface analysis of the alginate aerogels, the surface area and pore radius of the as-prepared aerogels were different. The pore sizes and pore volumes were measured directly from the analysis. Compared to the SA aerogel, introducing fillers improves the pore size of SAHA aerogel, meanwhile reducing the total BET surface area. It is well understood that, even if the pore size for SAHA aerogel is bigger than its counterpart, the number of total pores is less, thereby resulting in the decrease in total surface area [40].

### 3.2. Chemical Composition Analysis

#### 3.2.1. Fourier Transform Infrared Spectroscopy

**Figure 6:**
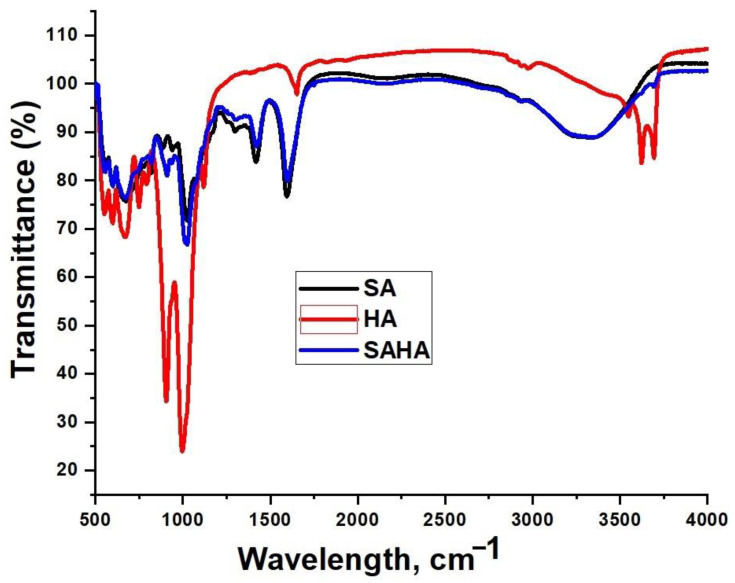
FT-IR spectra of the HA, SA, and SAHA aerogels.

The FT-IR spectra for the as-synthesized aerogels are shown in Figure 6. Both the spectra of alginate aerogel and the halloysite clay were consistent with the previous reports [41,42]. In the spectrum of halloysite clay, the absorption bands at 3695 and 3620 cm^−1^ were assigned to the stretching vibration of the O-H groups at the inner surface of halloysite. The presence of interlayer water was indicated by the vibration at 1652 cm^−1^. The 1111 cm^−1^ peak was assigned to the stretching mode of Si-O, while the band at 1030 cm^−1^ was caused by the stretching vibration of Si-O-Si. The band observed at 538 cm^−1^ was due to the vibration of Al-O-Si. The vibrations of the inner surface hydroxyl group at 912 cm^−1^ and Si-O-Si at 470 cm^−1^ confirmed the existence of these groups. The bands observed in the spectrum of halloysite showed no significant differences, since the majority of the bands were assigned to Si-O and Al-O bonds. For SA aerogel, a broad adsorption band at 3364 cm^−1^ corresponding to OH stretching, sharp peaks at 1608 and 1419 cm^−1^, which correspond to asymmetric and symmetric –COO− stretching vibration and –OH stretching can be seen. In the case of composite SAHA aerogel, most of the peaks corresponding to the HNTs are visible in the spectrum, which confirms the presence of them in the composite. Furthermore, the corresponding peaks of transmission bands at 3364 cm^−1^ of alginate and 3692 cm^−1^ of HNTs seems slightly shifted indicating the presence of hydrogen bonding in the composite aerogel. A slight shift in the peak around 1419 cm^−1^ of alginate to 1423 cm^−1^ can also be seen in the SAHA aerogel.

#### 3.2.2. Energy Dispersive X-ray Spectrometry (EDX) Analysis

**Figure 7:**
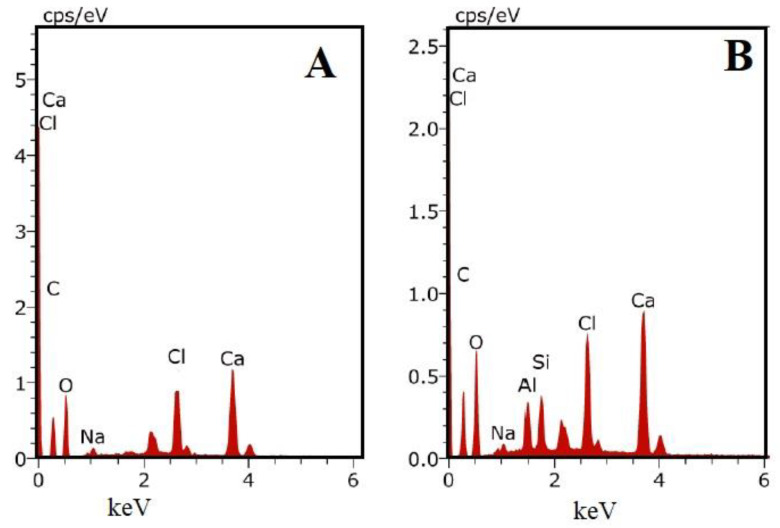
EDX spectra of (**A**) SA and (**B**) SAHA aerogels.

The elemental compositions of the as-prepared aerogels were semiquantitatively determined from EDX analysis (Figure 7). The SA aerogels contained only carbon, oxygen, and calcium elements, while in the SAHA aerogel, the presence of aluminum and silicon was also confirmed. The presence of calcium in both systems confirmed that the crosslinking process occurred in the alginate matrix.

### 3.3. Thermal Analysis of the Aerogels

#### Thermogravimetric Analysis

**Figure 8:**
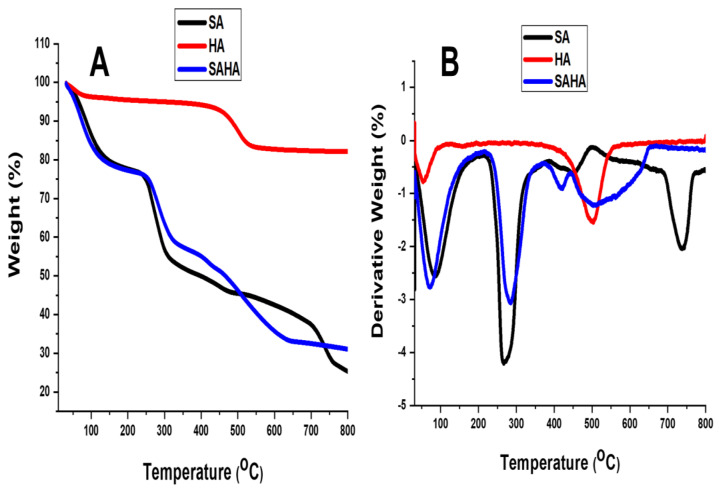
Thermal analysis curves (**A**) thermogravimetric analysis (TGA) and (**B**) differential thermal analysis (DTA) curves of halloysite clay (HA), SA, and SAHA.

The effect of halloysite clay on the thermal properties of the SAHA aerogel was analyzed using Thermogravimetric analysis (TGA) and Differential thermal analysis (DTA) curves. The resultant thermograms are shown in Figure 8. The TGA curve (Figure 8A) indicated a weight-decreasing pattern with respect to temperature, while the DTG curve (Figure 8B) represents the maximum temperature needed for the complete thermal degradation of the sample. The curve of halloysite clay revealed that one major mass loss occurred in the temperature range of 450–600 °C. An endothermic peak at 502 °C was observed, and this mass loss was assigned to the dehydroxylation of structural Al-OH groups of halloysite [43]. Another mass loss in the range 0–130 °C, indicated by an endothermic peak at 55 °C, corresponding to the loss of adsorbed water present on the surface and the interlayer, was also observed [44].

For aerogels, all the films exhibited a two-step thermal degradation pattern. The first stage of weight loss occurred at 70–90 °C and was due to the evaporation of loosely bound moisture; the second step of thermal degradation occurred at 190–280 °C and was attributed to the evaporation of glycerol and the thermal degradation of the biopolymer. The onset temperature for the second step of the thermal degradation of the composite films was similar to that of neat alginate films. The final residue remaining after thermal degradation at 800 °C (char at 800 °C) was 25.2% for the neat SA aerogel and 31.05% for SAHA. The increase in residue percentage was mainly due to the increased mineral content compared to that of clay.

The DTA graph of the sodium alginate aerogel (SA) shows four distinct peaks. An endothermic DTA peak at 90 °C was due to dehydration. The major degradation of sodium alginate occurred in the second temperature range from 180 to 500 °C (weight loss of approximately 50%), where loss of volatile components, rupture of chains, and fragmentation of sodium alginate occurred. This process was followed by an endothermic peak at 265 °C and an exothermic peak at 498 °C in the DTA curve. The final decomposition of sodium alginate and its fragmentation into monomers occurred in the third temperature range from 500 to 800 °C, and this process was followed by an endothermic peak with a maximum at 739 °C. These processes are followed by the conversion of fragments and monomer units into carbonate, a byproduct [17]. By incorporating halloysite into sodium alginate, the SAHA composite aerogel revealed an improved thermal degradation profile, in which the degradation temperature shifted to higher values. In this case, the major degradation between 180 and 500 °C was followed by an endothermic peak at 283 °C and an exothermic peak at 441 °C.

### 3.4. Oil/Water Wettability by Contact Angle Analysis

The surface characteristics of the developed SA and SAHA aerogels directly contribute to the wettability of water and oil, which was evaluated by measuring contact angles using a liquid drop shape analysis system. Due to the highly porous nature of the aerogels, both the water and oil droplets quickly spread and were absorbed by the aerogel. The contact angles of the water and oil droplets on the surfaces of both SA and SAHA aerogels were measured to be approximately 0°. Hence, the as-prepared SAHA aerogel was amphiphilic, probably due to the presence of a highly porous structure, surface roughness, and hydroxyl groups.

**Figure 9:**
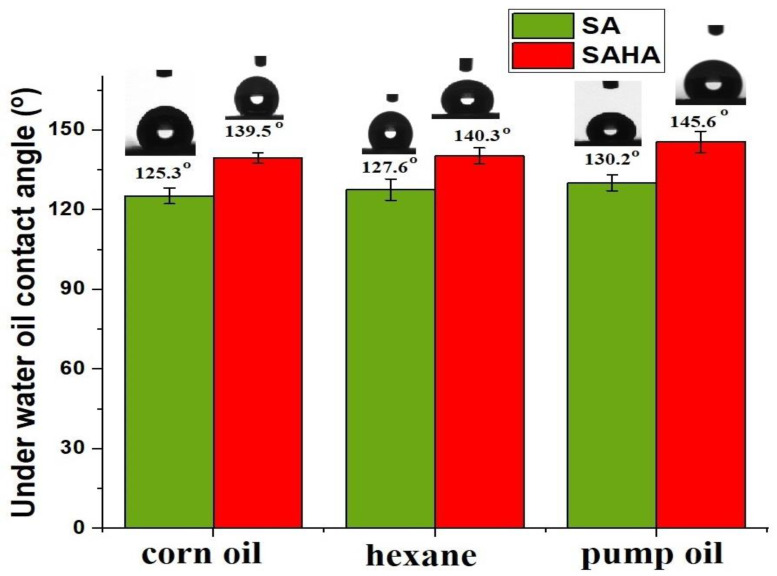
Underwater contact angles of various solvents for SA and SAHA aerogels.

The underwater oleophobicity of the as-prepared aerogels was tested by immersion in water. After complete soaking of the aerogels, the samples were tested against different oils, including corn oil, pump oil, and hexane. The results obtained were compared and are shown in Figure 9. The aerogels behaved differently when evaluating the underwater contact angle of oil. They no longer showed amphiphilic characteristics, and the surface rejected the oil droplets. The underwater contact angles of corn oil, hexane, and pump oil on the SA aerogel were approximately 125.3, 127.6, and 130.2°, respectively. The water molecules present in the water-soaked porous hydrophilic aerogels create a repulsive force against nonpolar solvents, which results in the underwater superoleophobic nature of the aerogels [45,46]. The SAHA aerogels showed improved water oil contact angles compared with the SA aerogels. The SAHA aerogel showed improved underwater oil contact angles of 139.5, 140.3, and 145.6° in the case of corn oil, hexane, and pump oil, respectively. The wettability of the aerogel surface was strongly dependent on the nature of the aerogel surface [38]. It can be concluded that the presence of tubular nanoclays in the composite aerogel improved the surface roughness and pore size of the aerogels, thereby improving the oil contact angle.

### 3.5. Oil/Water Separation Experiment

**Figure 10:**
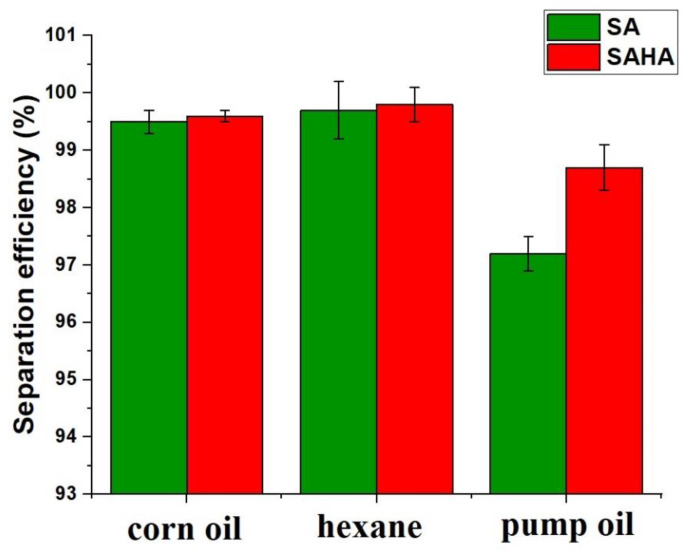
Oil/water separation efficiency of the SA and SAHA aerogels for different solvents.

Gravity-driven oil/water separation experiments using the as-synthesized SA and SAHA aerogels were performed using a homemade setup. When a 20 mL oil/water mixture (1:1) was poured through the filtration setup, clear water rapidly passed through the aerogel, while the red-dyed corn oil remained at the top of the aerogel filter. A clear filtrate was obtained, indicating the efficiency of the separation. The separation efficiencies of the aerogels for each oil were calculated using Equation (1) and are shown in Figure 10. The oil/water separation efficiencies of corn oil, hexane, and pump oil for SA aerogel were found to be 99.5, 99.7, and 97.2%, respectively. The separation efficiency for pump oil was low because of the density of the oil compared to other counterparts in this study. Through the incorporation of halloysite clay nanotubes, the SAHA aerogels showed improved separation efficiencies for corn oil, hexane, and pump oil of 99.6, 99.8, and 98.7%, respectively.

Moreover, the presence of hydrogen bonding in the SAHA aerogel prevented oil from penetrating the pores in a water environment, thereby improving the oil intrusion pressure [11]. The maximum height of the oil column withstood by the SAHA aerogel was 16.4 cm, which was approximately 2.1 cm higher than that of the SA aerogel (14.3 cm). The oil intrusion pressure was calculated using Equation (2) and was found to be ~1.23 and ~1.49 kPa for the SA and SAHA aerogels, respectively. Hence, the presence of halloysite clay improved the oil intrusion pressure of the SAHA aerogel up to 20% when compared to the SA aerogel. The water flux calculated for the SAHA aerogel was 6.78 L m^−2^ s^−1^, which was higher than the 3.42 L m^−2^ s^−1^ for the SA aerogel.

**Figure 11:**
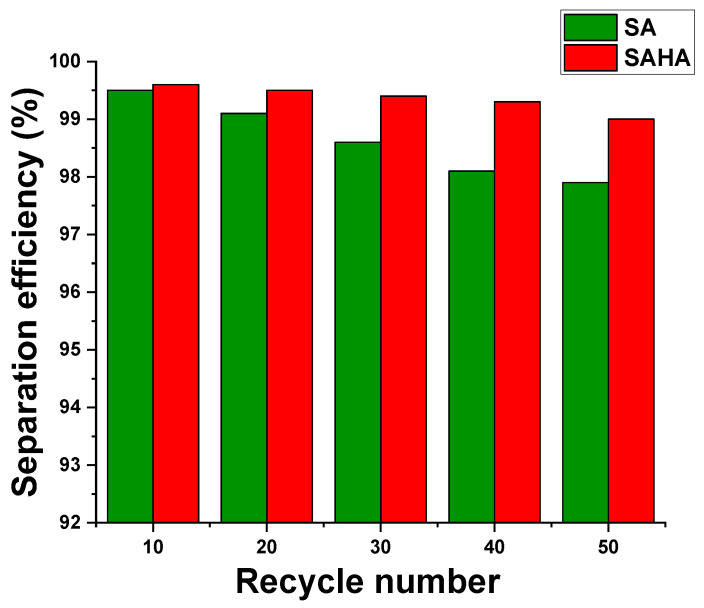
Separation efficiency versus recycle numbers (corn oil/water mixture as sewage).

The reusability of the as-synthesized SAHA aerogel was also studied by repeating the oil/water separation experiments with the aerogel membrane, and the result is shown in Figure 11. In the case of the SA aerogel, with increasing cycle number, the separation efficiency decreased from 99.5 to 97.9% after 50 reuses. The SAHA aerogel only showed a slight decrease (0.6%), indicating its highly stable oil–water separation ability in aqueous environment.

## 4. Conclusions

This study explained the synthesis and systematic characterization of alginate–halloysite nanocomposite aerogels using an environmentally benign protocol including a two-step ionic crosslinking process followed by freeze-drying. The as-synthesized aerogels showed a 3D porous structure with high surface roughness. The aerogel showed an amphiphilic character towards water and oil in air, while it exhibited underwater superoleophobicity. The SAHA aerogel could separate various oil/water mixtures more efficiently (up to 99.8%) when compared with SA aerogel and could be reused for up to 50 cycles. The use of biopolymers and environmentally friendly fillers for designing materials for wastewater treatment is in high demand. This kind of aerogel can evolve to be an ideal candidate for the effective cleaning of oil spills in aquatic environments.

## Figures and Tables

**Table 1:** ***Brunauer-Emmett-Teller*** (BET) surface area, pore size, and pore volume of the aerogels.

Sample	BET Surface Area (m^2^/g)	Desorption Average Pore Radius (BJH) (nm)	*t*-Plot Micropore Volume Cc/g
SA aerogel	12.1	10.6	0.035
SAHA aerogel	2.9	37.6	0.0115
